# Genetic and functional analysis of *ZMIZ1* in neurodevelopmental disorder with dysmorphic facies and distal skeletal anomalies (NEDDFSA): insights from muscle cells and signaling pathways

**DOI:** 10.1038/s41390-025-04612-x

**Published:** 2025-12-07

**Authors:** Shan Li, Dafei Zhou, Yuyang Han, Chao Feng, Xuemin Lv, Gang Fu, Xing Wei, Chengai Wu, Chaoji Yu, Zheng Yang

**Affiliations:** 1 Department of Molecular Orthopaedics, National Center for Orthopaedics, Beijing Research Institute of Traumatology and Orthopaedics, Beijing Jishuitan Hospital, Capital Medical University, Beijing, China; 2 Department of Pediatric Orthopaedics, National Center for Orthopaedics, Beijing Jishuitan Hospital, Capital Medical University, Beijing, China; 3 Beijing Huanuo Aomei Gene Biotech Co. Ltd, Beijing, China

## Abstract

**Background:**

Mutations in the *ZMIZ1* gene have been implicated in neurodevelopmental disorder with dysmorphic facies and distal skeletal anomalies (NEDDFSA). However, the underlying cellular and physiological mechanisms remain poorly understood.

**Methods:**

Exome sequencing was performed to identify candidate variants. qPCR, Western blot, immunofluorescence, CCK-8, and wound-healing assays were employed to assess gene function in human skeletal muscle cells (HSkMCs). RNA-seq and co-immunoprecipitation coupled with mass spectrometry (Co-IP/MS) were used for transcriptomic and interactome profiling.

**Results:**

Here, we identified a novel de novo missense variant c.910G>C (p.A304P) in *ZMIZ1* in a patient with NEDDFSA. The p.A304P variant significantly increased *ZMIZ1* mRNA and protein expression levels and altered its subcellular localization. Functional assays demonstrated enhanced proliferation and migration in HSkMCs expressing the mutant *ZMIZ1*. qPCR validation revealed significant dysregulation of key genes in the TGF-β1 signaling pathway. Transcriptome analysis identified the cytokine-cytokine receptor interaction pathway as the most significantly enriched pathway. Additionally, we identified a novel interaction between ZMIZ1 and the transcription factor GTF2I.

**Conclusion:**

Our study identifies a novel likely pathogenic variant in *ZMIZ1* associated with NEDDFSA. These findings provide new insights into the cellular and physiological mechanisms underlying NEDDFSA, highlighting ZMIZ1’s role as a regulatory hub in multiple signaling pathways.

**Impact:**

- Our study identifies a novel likely pathogenic variant in the *ZMIZ1* gene associated with NEDDFSA, the second in China, and demonstrates its functional impact on cellular processes and signaling pathways, particularly in muscle cells. This finding expands the known *ZMIZ1* mutation spectrum and provides the first functional evidence of its role in muscle cells, highlighting its potential as a regulatory hub in multiple pathways beyond the nervous system. These results offer new insights into the cellular mechanisms underlying NEDDFSA, especially in non-neurological tissues, and may facilitate the development of targeted therapies for related disorders.

## Introduction

Neurodevelopmental disorder with dysmorphic facies and distal skeletal anomalies (NEDDFSA, OMIM #618659) is a genetically and clinically heterogeneous syndromic intellectual disability characterized by generalized neurodevelopmental impairment, early-onset muscular hypotonia, microsomatia, delayed language development, behavioral abnormalities and supplementary shared characteristics including joint hypermobility or joint contracture, eye anomalies, and acral skeletal malformations of extremities.^1^ NEDDFSA results from heterozygous variants in the *ZMIZ1* gene (*zinc finger MIZ-containing 1*; OMIM #607159), inherited in an autosomal dominant manner.

To date, 27 cases of *ZMIZ1* variants have been reported,^1–7^ showing a wide range of systemic symptoms including developmental delay/intellectual disability, as well as cardiac, skeletal, and genitourinary malformations, combined with ocular abnormalities. NEDDFSA cases caused by *ZMIZ1* variants remain rare, particularly in East Asia populations. The first Chinese case was reported by Lu et al.,^5^ and this study describes the second confirmed *ZMIZ1* variant in the Chinese population. Further case identification and mechanistic studies are needed to improve the understanding of NEDDFSA pathogenesis and clinical management.

The *ZMIZ1* gene (NM_020338.3) maps to chromosome 10q22.1-q22.3 and comprises 21 exons, encoding a 130 kDa protein consisting of 1067 amino acids.^8,9^ The ZMIZ1 protein comprises several distinct domains, including a Zmiz1 N-terminal tetratricopeptide repeat domain (Zmiz1_N, spanning residues 8–100), a Med15 domain (covering residues 184–557), a nuclear localization signal (NLS, located between residues 697 and 711), a transactivation domain (TAD, extending from residues 837–1067), and a SP-RING zinc finger domain (SP-RING_ZMIZ, ranging from residues 739–786).^10^ *ZMIZ1* encodes a protein that belongs to the PIAS-like family, known for its interaction with nuclear hormone receptors. This protein contains a highly conserved Msx-interacting zinc finger domain (MIZ), which plays a significant role in SUMOylation processes and facilitates protein-protein interactions.^10^ *ZMIZ1* indirectly regulates numerous transcription factors, including TP53, NOTCH1, and androgen receptor (AR), by functioning as a coactivator of transcription regulator. The expression levels of *ZMIZ1* were detected in all tissue types and specific brain regions.^10^ Notably, the heart, ovary, and brain exhibited the highest levels of *ZMIZ1* expression, while the amygdala displayed the highest expression levels within the brain.^11^ Although musculoskeletal tissues were not extensively profiled in earlier studies, recent evidence from the Genotype-Tissue Expression (GTEx) project confirms high *ZMIZ1* expression in skeletal muscle (median TPM = 26.5 in healthy adult).^12^ The *ZMIZ1* gene has been reported to be important in chondrogenesis or cartilage-related diseases.^13^ These findings imply that the *ZMIZ1* gene may play an important role in the function of joint or muscle cells, providing biological rationale for investigating *ZMIZ1* variants in joint/cellular phenotypes.

*ZMIZ1* regulates dendritic development and neuronal synapse development through chromatin remodeling in the brain.^14^ *ZMIZ1* pathogenic variants electroporated into mouse cortices (E14.5) *in utero* led to impaired neuronal position as a result of affected ventricular zone (VZ) progenitors.^1^ Hence, *ZMIZ1* variants were considered to be the underlying genetic determinants responsible for NEDDFSA. However, in addition to the nervous system, there is little research on how *ZMIZ1* causes abnormal development of other body regions, such as joints.

Despite these findings, the cellular and physiological mechanisms underlying NEDDFSA remain poorly understood, particularly in non-neurological tissues such as muscles and joints. The discovery of more cases and further study of ZMIZ1’s mechanisms are essential for a comprehensive understanding of the disorder. In our study, a novel ZMIZ1 variant in a Chinese patient with NEDDFSA was investigated. We provided novel insights into the cellular and physiological consequences, including its unprecedented effects on muscle cells. Our findings reveal the impact of the ZMIZ1 variant on cellular proliferation, migration, and signaling pathways, highlighting its role as a regulatory hub in multiple pathways essential for tissue development and homeostasis.

## Materials and methods

### Blood sample collection

Written informed consent for genetic analysis and publication of data was obtained from all participants. The experimental workflow used in this study is shown in Fig. S1. This study complied with the Helsinki Declaration and was approved by the Ethics Committee of Beijing Jishuitan Hospital, Capital Medical University. Peripheral blood samples (4–6 mL) were collected in EDTA tubes. Genomic DNA of the patient and his parents was then isolated using the QIAamp DNA Blood Midi Kit (Qiagen, Hilden, Germany) and quantified using a Nanodrop 2000 spectrophotometer (Thermo Scientific).

### Whole exome sequencing (WES) and data analysis

Whole-exome sequencing (WES) was performed in the proband (II-1) by Beijing Huanuo Aomei Gene Biotech Co., Ltd. We used the IDT kit for a full exome probe to capture, enrich, and control the quality of the enriched library. Quality control of sequencing data was performed then aligned to the human reference genome (GRCh37/hg19) using the Burrows-Wheeler Aligner software. After that, the GATK software was used for variable heterotopic point detection (SNP/dup/indel). Based on the self-developed software, Clinvar, ExAC, 1000 Genome, and other public databases, the HGMD commercial database and the self-built local database of the Chinese population, were called for gene function analysis. The American College of Medical Genetics (ACMG) standards and guidelines were applied to determine the pathogenicity of each variant.^15^ The detailed quality control of sequencing data is presented in Table S1. These methods align with our previous study.^16^ Sanger sequencing with specific primers was conducted to confirm the identical variants of the proband. All primers were designed using the online tool Primer 3.

### Molecular modeling and structural analysis

The amino acid sequence of the ZMIZ1 was obtained from the NCBI Protein database (FASTA format). Multiple sequence alignments with different species and conservation analysis were performed using software MEGA. 3D structures of wild-type and mutant ZMIZ1 proteins were generated by homology modeling using the SWISS-MODEL server (http://swissmodel.expasy.org/). The interactions between amino acid and adjacent residues were simulated with the PyMOL (Schrödinger, LLC, http://www.pymol.org/).

### Cell culture

Purchased commercial HEK293T cells were cultured in Dulbecco’s modified Eagle’s Medium (DMEM, Gibco) containing 10% fetal bovine serum (FBS, Gibco), L-glutamine and 1% penicillin-streptomycin at 37 °C and at 5% CO2 atmosphere. Human skeletal muscle cells (HSkMCs) were purchased from ScienCell (California) and maintained in dedicated growth medium (Cell Applications) containing 10% FBS, 1% NEAA, 1% insulin-transferrin-selenium (ITS), and 1% antibiotic-antimycotic (Gibco) at 37 °C in a humidified atmosphere containing 5% CO_2_. All cell lines were routinely tested for mycoplasma contamination. HSkMCs at passage ≤5 were used for experiments.

### Plasmids construction and transfection of HSkMCs and HEK293T Cells

Coding sequences (CDS) sequences of wild-type and c.910G>A variant of human *ZMIZ1* (NM_020338.3) synthesized commercially. The CDS sequences were cloned into pcDNA3.1-3xFlag (LMAI Bio Co., Ltd) and VP001-CMV-MCS-3xFlag-EF1-ZsGreen-T2A-Puro (Anhui General Biology Co., Ltd) vectors. Sequences of all expression vectors were confirmed by Sanger sequencing with primer ZMIZ1-910-F: 5′-ACACCAACCAGCCTCCCGGCT-3′. HEK293T cells were transiently transfected with pcDNA3.1-3xFlag-ZMIZ1 plasmids (wild-type/mutant) using JetPRIME transfection reagent (Polyplus), with empty vector pcDNA3.1-3xFlag as a negative control. After transfection for 48 h, protein of the cells was collected for the follow-up experiments.

### Quantitative real-time PCR (qPCR)

Total RNA was extracted from transfected HSkMCs (Vector, ZMIZ1-WT, and ZMIZ1-Mutant groups; *n* = 3 biological replicates per group) using Trizol (Invitrogen, Carlsbad, CA), then reverse transcribed into cDNA templates using HiScript III All-in-one RT SuperMix Perfect for qPCR (Vazyme, Nanjing, China), according to the manufacturer’s protocol. qPCR was performed with AcceQ Universal SYBR qPCR Master Mix (Vazyme, China) on an ABI PRISM 7500 Real-Time System. Primer sequences are listed in Table S2. Relative mRNA expression was calculated via the 2^−ΔΔCt^ method normalized to GAPDH. Data represent mean ± SD. Significance was determined by Student’s *t* test (*P* < 0.05).

### Western blotting (WB) analysis

Transfected HSkMCs and HEK293T cells were lysed in RIPA buffer and centrifuged at 12,000 ×* g* at 4 °C for 10 min, total protein was collected. And Nuclear and cytoplasmic fractions were isolated using a commercial extraction kit (Invent, SC-003) according to the manufacturer’s protocol. Equal amounts of protein (20 μg) from different samples were separated by SDS-PAGE (4–12% gradient), and then transferred onto PVDF membrane. The membranes were then incubated with primary antibodies against anti-FLAG (ab205606, Abcam) and GAPDH (ab181602, Abcam), LaminB1(proteintech, 66095-1-Ig) and β-actin (proteintech, 20536-1-AP), at 4 °C overnight. The secondary anti-rabbit IgG (H + L) antibody (5220-0336, KPL) was added to the membranes, followed detecting by the ECL Plus reagent (Millipore), and images were recorded in the Gel Imaging System (Bio-Rad Laboratories).

### Immunofluorescence staining

After transfection for 48 h, HSkMCs and HEK293T cells were fixed with 4% paraformaldehyde and permeabilized with 0.2% Triton X-100 for 20 min and blocked with 1% BSA in PBS for 60 min, then stained with anti-FLAG (ab205606, Abcam) incubated at 37 °C for 2 h. Alexa Fluor® 594 goat anti-rabbit IgG (H + L) secondary antibodies (ZF-0516, ZhongShanJinQiao) were incubated at room temperature without light for 60 min and counterstained with DAPI for 5 min. After washing with PBS for 3 times, coverslips were mounted and imaged using a 60× objective lens under an OLYMPUS fluorescence microscope.

### Lentivirus packaging and transduction

HEK293T cells were used to package lentiviral particles. Lentiviral vectors encoding full-length human ZMIZ1-WT (VP001-CMV-MCS-3xFlag-EF1-ZsGreen-T2A-Puro) or ZMIZ1-Mutant, along with empty vector plasmid as a negative control were synthesized by General Biology (Anhui) Co., Ltd. The lentivirus transfer plasmid was transfected into HEK293T cells with packaging plasmids pLP-VSVG, pLP1, and pLP2 using PolyJet Reagent (Cat. #SL100688, SignaGen) to generate lentiviral particles. 48 h and 72 h later, the lentivirus-containing supernatants were collected, filtered through a 0.45-μm filter, and concentrated by ultracentrifugation. The lentivirus particles (MOI = 5) were added when the HSkMCs cell density reached 50–60% confluence. In the experiment, transfection was divided into three groups, including ZMIZ1-WT group, ZMIZ1-Mutant group, and Vector/BLANK group. The overexpression of ZMIZ1 was confirmed by WB.

### Ethynyl deoxyuridine (EdU) assay

Transduced HSkMCs cells were divided into 3 groups: (i) Vector group (BLANK); (ii) ZMIZ1-WT group; (iii) ZMIZ1-Mutant group. At 24 h after infection, the media was removed, the cells were washed by PBS, complete media was added to the culture. HSkMCs were transfected for 48 h, treated with pancreatin, and then cultured overnight under standard conditions for the EdU assay. A BeyoClick™ EdU-594 Cell Proliferation Kit (Beyotime Biotech) was used for the EdU assay. Briefly, the EdU reagent was dissolved directly into the culture medium, resulting in a final concentration of 10-μM. After incubation for 2 h, the HSkMCs cells were fixed with paraformaldehyde for 15 min and stained with Click Additive Solution in the dark. Thereafter, DAPI staining was used to counterstain nuclei, and EdU-positive cells were photographed and counted in random fields.

### Scratch wound healing assay

Transfected HSkMCs cells were seeded in 6-well culture plates. At 24 h after infection, the medium was removed, the cells were washed with PBS, and complete medium was added to the culture. HSkMCs cells were divided into 3 groups: (i) Vector group (BLANK); (ii) ZMIZ1-WT group; (iii) ZMIZ1-Mutant group. Next, 200-µL pipette tips were used to create scratch wounds around each well’s diameter. Photos were taken, and cell migration was recorded at 0 h under a microscope using 10× objective lens. Finally, the medium was replaced with fresh medium and wound healing was measured and photographed under a microscope at 24 h and 48 h within the wound line and the healing area was calculated.

### Transwell assay

Transfected HSkMCs cells were seeded in 6-well culture plates. At 24 h after infection, the media was removed, complete media was added to the culture. HSkMCs cells were divided into 3 groups: (i) Vector group (BLANK); (ii) ZMIZ1-WT group; (iii) ZMIZ1-Mutant group. HSkMCs were transfected for 48 h, treated with pancreatin. Then, cultured transwell chambers (Corning Life Sciences, Corning, NY) and Matrigel (BD Biosciences, Franklin Lakes, NJ) were used to conduct the transwell assays. The cells were collected 24 h and 48 h after infection. The cells were fixed with anhydrous methanol, stained with 0.1% crystal violet, and photographed.

### Transcriptome sequencing

RNA samples from transfected HSkMC experimental groups underwent library construction. Paired-end libraries were synthesized using the TruSeq® RNA Sample Preparation Kit (Illumina) according to the manufacturer’s instructions. Briefly, poly(A)-containing mRNA molecules were purified using poly(T) oligo-attached magnetic beads. The mRNA was then fragmented into small pieces using divalent cations at 94 °C for 8 min. The cleaved RNA fragments were copied into first-strand cDNA using reverse transcriptase and random primers followed by second-strand cDNA synthesis using DNA polymerase I and RNase H. The cDNA fragments then underwent end repair, the addition of a single adenosine, and then ligation of the adapters. Next, the products were purified and enriched using PCR to create the final cDNA library. Purified libraries were quantified using a Qubit® 2.0 fluorometer (Life Technologies) and validated using an Agilent 2100 bioanalyzer (Agilent Technologies) to confirm the insert size and calculate the concentration. Clusters were generated by cBot with the library diluted to 10 pM and then sequenced on an Illumina NovaSeq 6000 (Illumina). The cDNA library construction and sequencing were performed at Shanghai Biotechnology Corporation.

### Functional analysis of differentially expressed genes (DEGs)

Raw sequencing reads were preprocessed by removing sequencing adapters, short-fragment reads, and other low-quality reads. Clean reads were aligned to the human reference genome GRCh38 using HISAT2, allowing up to two mismatches. After alignment, StringTie was used with reference annotation to generate fragments per kilobase of transcript per million mapped reads (FPKM) values for known gene models. DEGs were identified using edgeR. The *P* value set as the significance threshold for multiple tests was determined by the false discovery rate (FDR) correction. Fold changes were calculated based on the FPKMs for each sample. Differentially expressed genes were defined as with an adjusted *P* value (padj) ≤ 0.05 and a fold change ≥ 2. Functional enrichment analysis of DEGs was conducted using the Gene Ontology (GO) and KEGG pathway databases.

### Co-immunoprecipitation and mass spectrometry (Co-IP/MS)

Flag-tagged full-length ZMIZ1-WT and ZMIZ1-Mutant were cloned into the pcDNA3.1 (+)-Flag eukaryotic expression vectors. The plasmids were then transfected into HEK293T cells. Cells were harvested 48 h after transfection and lysed in lysis buffer (1% NP-40, 20 mM Tris-HCl [pH 7.5], 150 mM NaCl, 5 mM EDTA, 1 mM Na3VO4, 0.25% sodium deoxycholic acid, and complete protease inhibitor cocktail [Roche]) on ice for 30 min. After centrifugation at 12,000 rpm (11,800 × *g*) for 15 min at 4 °C, supernatants were collected and incubated with Protein A/G Sepharose beads (SC-2003; Santa Cruz) coupled to anti-Flag (Sigma, F1804) with 25 μg overnight at 4 °C. Beads were washed three times with high-salt wash buffer (1% Triton X-100, 20 mM Tris-HCl [pH 7.5], 500 mM NaCl, 10% glycerol, 2 mM EDTA, 1 mM Na3VO4, and a complete protease inhibitor cocktail [Roche]), followed by another three times with low-salt wash buffer (1% Triton X-100, 20 mM Tris-HCl [pH 7.5], 150 mM NaCl, 10% glycerol, 2 mM EDTA, 1 mM Na3VO4, and complete protease inhibitor cocktail [Roche]). Proteins were eluted by boiling with 300 μl SDS sample buffer for 10 min. ZMIZ1-interacting proteins were identified by mass spectrometry.

### Statistical analysis

All data are presented as mean ± SD. Differences among groups were evaluated using one-way analysis of variance (ANOVA) or unpaired Student’s *t* test. All statistical analyses were performed using the GraphPad Prism version 9.0 (GraphPad Software, Inc.). A *P* ≤ 0.05 was considered statistically significant. *P < 0.05; **P < 0.01; ***P < 0.001; ****P < 0.0001.

## Results

### Identification of a novel ZMIZ1 variant in a NEDDFSA patient

**Fig. 1:**
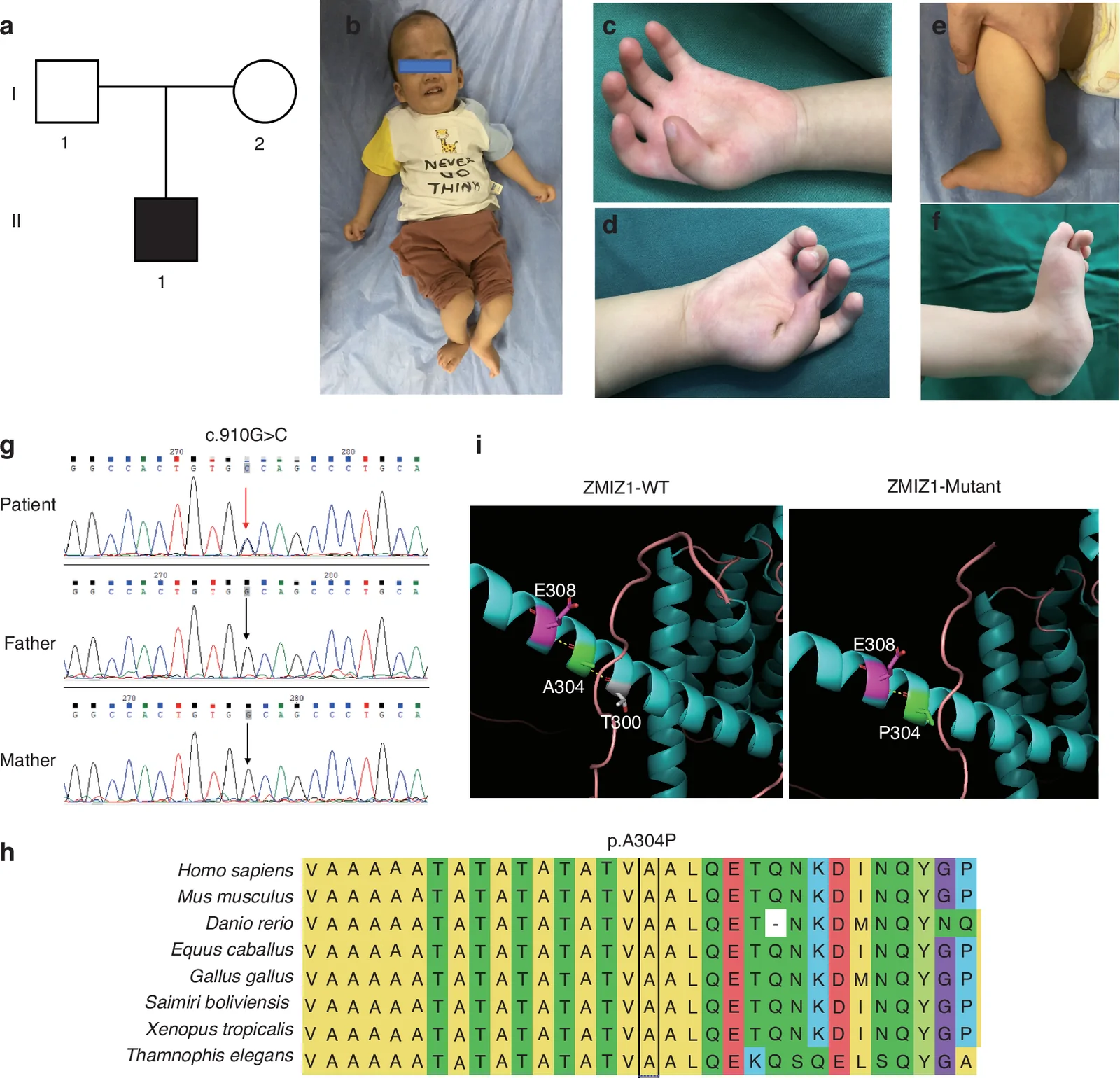
Pedigree, clinical features in patient and identified a novel mutation in *ZMIZ1.* **a** Black arrow denotes the proband. Open circles and squares represent unaffected females and males. Filled circles and squares represent affected members. **b**–**f** Clinical presentation of Patient (II1) showing bilateral auricular deformity, hand contractures, congenital vertical talus, and developmental delay. **g** Sequence chromatograms from Sanger sequencing analysis of the *ZMIZ1* gene showing a heterozygous mutation c.910G>C(p.A304P) in patient and his parents is normal. **h** Conservation analysis of Ala304 residue (highlighted in box) in *ZMIZ1* across different species. **i** Three-dimensional structure of the missense mutation site Ala304 in *ZMIZ1*. A hydrogen bond between Ala304 and Thr300 disrupted by the mutation p.Ala304Pro.

The patient (II1) was born to nonconsanguineous parents of normal phenotype (Fig. 1a). Clinical manifestations of patient 1(II1) (11 months old, height 70 cm, Z-score = −2.52) are small stature, bilateral auricular deformity (Fig. 1b), bilateral hand contractures (Fig. 1c–e), congenital vertical talus (Fig. 1f), developmental delay, hearing impairment, and bilateral cryptorchidism.

WES identified rare variants in the proband (Table S3). ZMIZ1, a rare variant linked to neurodevelopmental disorders, was identified in the patient during the phenotype-driven analysis of rare variant association. The patient’s DNA was amplified using ZMIZ1-E1-F/R primers (Table S2), revealing a heterozygous missense variant c.910G>C (p.A304P) (Fig. 1g). This variant was absent from major genetic databases (ExAC, 1000 g, 1000g_EAS and gnomAD_ ALL) (Table S4).

Conservation analysis revealed that the p. Ala304 residue is evolutionarily conserved in zinc finger MIZ-containing 1 protein (Fig. 1h), suggesting its critical role in preserving the structural integrity or functional activity of the protein across different species. The missense mutant ZMIZ1 protein exhibited modifications in its structure (Fig. 1i). In the wild-type protein, Ala304 is predicted to establish hydrogen bonds with Glu308 and Thr300. However, the variant induces steric hindrance effect, and breaks a hydrogen bond at Thr300, potentially modifying its biological activity (Fig. 1i). Bioinformatics analysis revealed p.A304P variant in ZMIZ1 is damaging by multiple protein harmfulness prediction software (Table S4). The bioinformatics analysis yielded further understanding regarding the potential pathogenicity of the variants. The variant was classified as “Likely Pathogenic” according to ACMG/AMP guidelines^15^ with evidence codes summarized in Table S5.

### *ZMIZ1* variant alters protein expression and nuclear localization

**Fig. 2:**
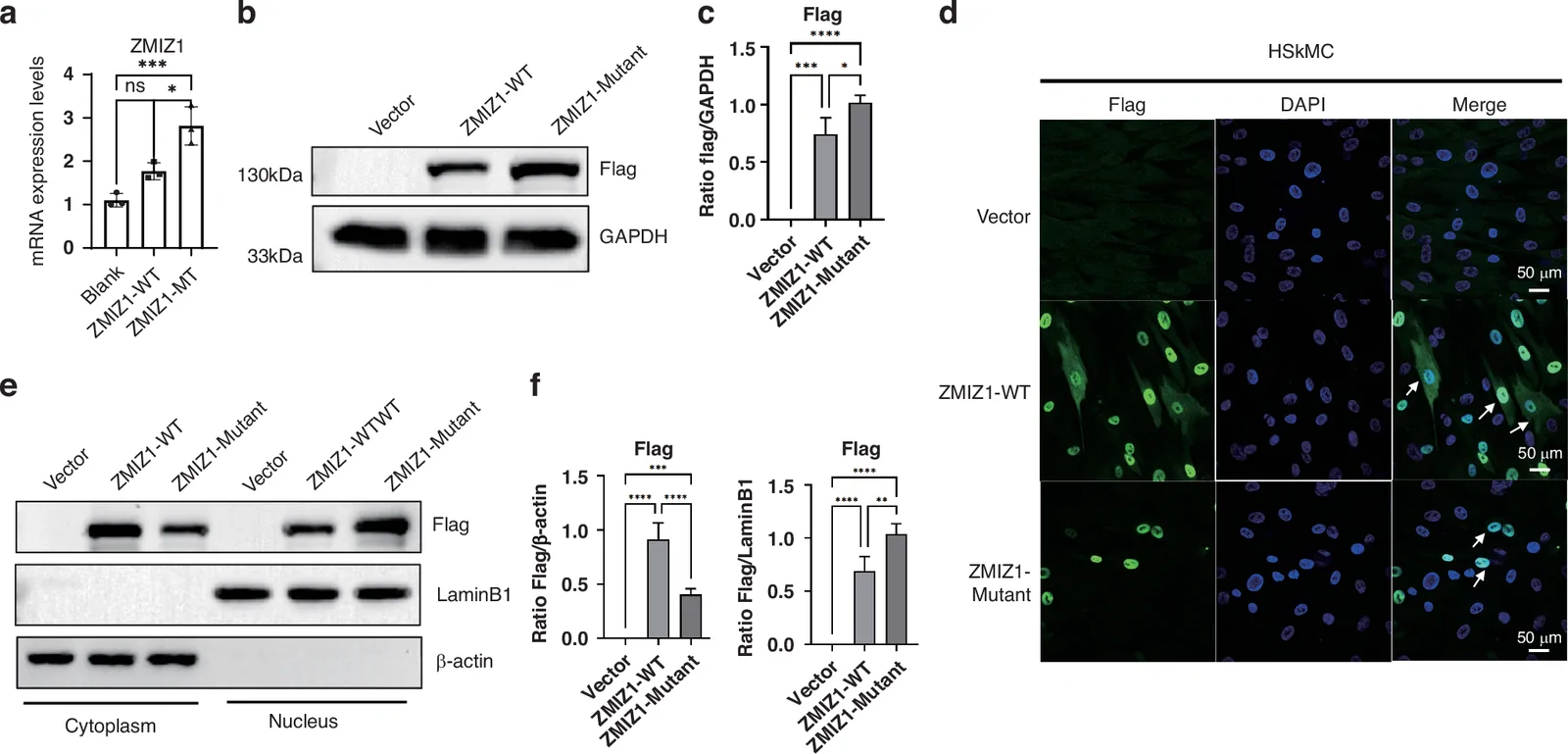
Function and expression of *ZMIZ1* variant p.A304P in HSkMCs. **a** Expression of *ZMIZ1* mRNA was assessed by qRT-PCR. p.A304P *ZMIZ1* was significantly upregulated compared to WT in vitro, as indicated by RT-qPCR. **b**, **c** The protein expression levels of ZMIZ1 were measured by WB. ZMIZ1-mutant was significantly upregulated compared to ZMIZ1-WT in vitro, as indicated by WB. **d** Cellular localization of *ZMIZ1* in cells transfected with plasmids pcDNA3.1, pcDNA3.1-*ZMIZ1*-WT, or pcDNA3.1-*ZMIZ1*-mutant. Cells were stained with anti-Flag antibody (green). Nuclei were stained with DAPI (Blue). ZMIZ1-WT was diffusely expressed in the nucleus and cytoplasm, while ZMIZ1-mutant was expressed mainly in the nucleus. Scale bar: 50 μm. **e**, **f** WB analysis of ZMIZ1 protein expression in nuclear and cytoplasmic fractions. Cellular fractionation confirms enhanced nuclear accumulation of mutant ZMIZ1 (LaminB1 nuclear marker; β-actin cytoplasmic marker).

To evaluate the pathogenicity of the p.A304P variant, functional analyses were performed assessing both the expression levels and the subcellular localization in HSkMCs (Fig. 2) and HEK293T cells (Fig. S2). Reverse transcription-qPCR (RT-qPCR) demonstrated increased ZMIZ1 mRNA expression in cells expressing the p.A304P mutation (ZMIZ1-Mutant) compared to the wild-type (ZMIZ1-WT) (Fig. 2a and Fig. S2A). Consistently, WB analysis revealed elevated protein expression in ZMIZ1-Mutant transfected HSkMCs (Fig. 2b, c) and HEK293T cells (Fig. S2B, C). In cells transfected with an empty vector, minimal immunofluorescence signal was observed along the cell membrane. Immunofluorescence of ZMIZ1-WT showed localization primarily at the plasma membrane, with additional signal in the cytoplasm and nucleus (Fig. 2d and S2D). Conversely, in cells transfected with the p.Ala304Pro variant, signals were restricted to fewer cells and predominantly localized to nuclei (Fig. 2d, e and Fig. S2D, E). Both WB and immunofluorescence results confirmed increased ZMIZ1 expression in the mutant, consistent with RT-qPCR data (Fig. 2 and Fig. S2). Nuclear-cytoplasmic fractionation by WB confirmed that both wild-type and mutant ZMIZ1 proteins were detectable in cytoplasmic and nuclear fractions. However, compared to the wild-type protein, the mutant ZMIZ1 showed increased nuclear localization and decreased cytoplasmic localization (Fig. 2e, f). Notably, an additional ~110 kDa band was observed in transfected HEK293T cells, but not in HSkMCs, suggesting cell-type-specific processing of mutant ZMIZ1. This discrepancy may result from differential proteolytic activity or distinct subcellular trafficking. Collectively, these findings demonstrate that the p.A304P variant alters subcellular localization and enhances ZMIZ1 expression.

### *ZMIZ1* variant promotes proliferation and enhances migration of HSkMC

**Fig. 3:**
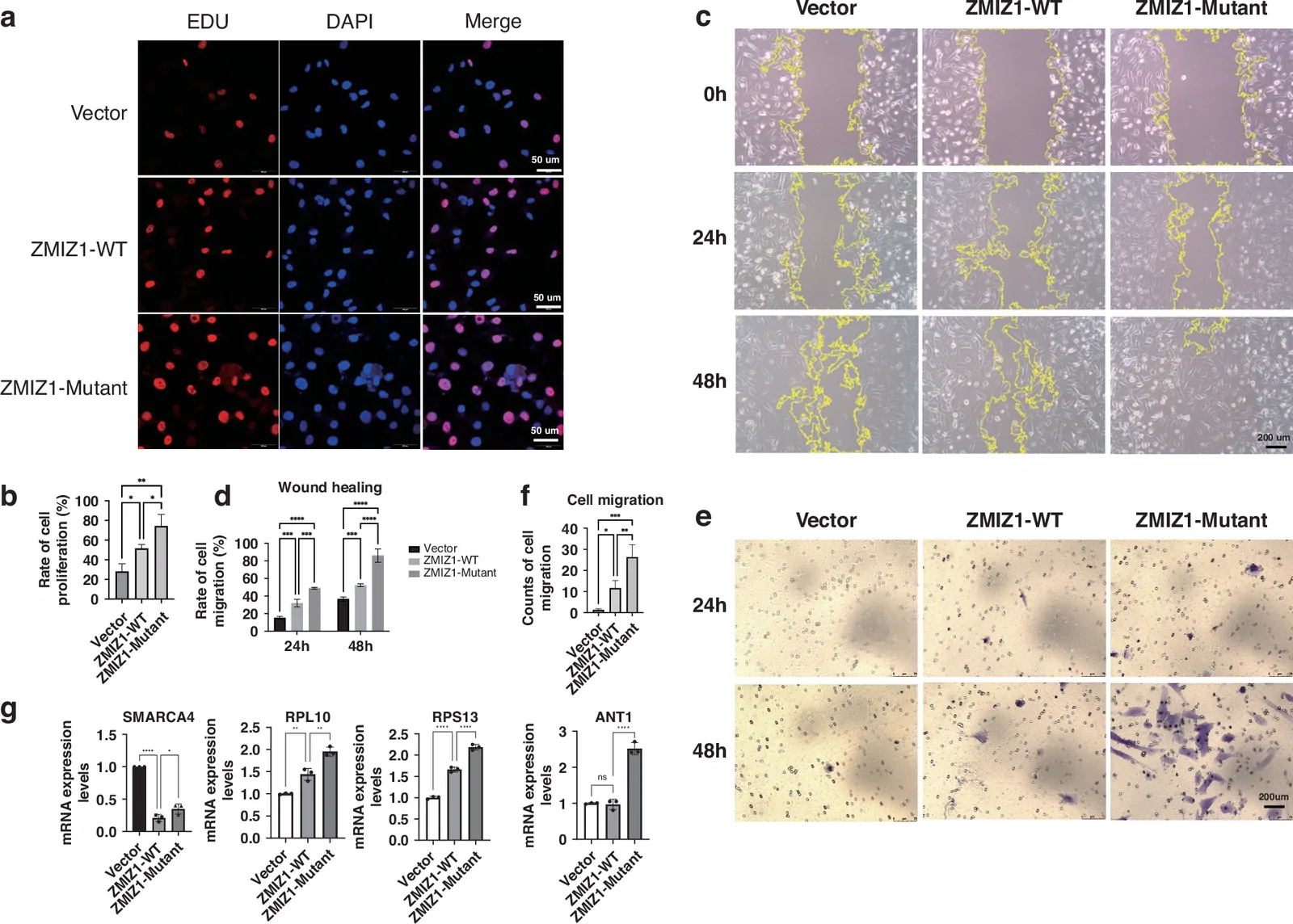
ZMIZ1-Mutant enhances HSkMCs proliferation, invasion and migration. **a**, **b** In comparison to the wild type, EdU incorporated cell proportion was significantly increased in the p.A304P variant. Scale bar: 50 μm. **c**, **d** The scratch experiment showed that the healing speed of scratches in the ZMIZ1*-*Mutant group was faster than the ZMIZ1*-*WT. Scale bar: 200 μm. **e**, **f** The transwell migration assay proved that the ability of cell migration was boosted in ZMIZ1*-*Mutant group in HSkMC cells. Scale bar: 200 μm. **g** Upregulation of SMARCA4, RPL10, RPS13, and ANT1 genes in mutant-transfected HEK293T.

Although ZMIZ1 mutations are associated with neurodevelopmental disorders, they do not fully explain the joint abnormality in NEDDFSA patients. To investigate whether the ZMIZ1 mutation affects muscle cell development and contributes to joint contractures, we assessed the impact of the p.Ala304Pro variant on muscle cells for the first time. Compared to ZMIZ1-WT, cells expressing the p.Ala304Pro mutant (ZMIZ1-Mutant) exhibited a significantly higher proportion of EdU-incorporated cells, indicating enhanced proliferation (Fig. 3a, b). Consistent with this, scratch assays revealed accelerated wound closure in ZMIZ1-Mutant cells (Fig. 3c, d). Notably, proliferation was substantially increased at 24 and 48 h. Transwell migration assay further confirmed enhanced cell migration capacity in the ZMIZ1-Mutant group within HSkMC cells (Fig. 3e, f). These results demonstrate that p.A304P variant promotes proliferation, migration, and invasion i*n vitro*.

Furthermore, we also detected the expression levels of proliferation-related genes in HEK293T cells, like chromosome remodeling gene *SWI/SNF-related, matrix-associated, actin-dependent regulator of chromatin, subfamily A, member 4* (*SMARCA4*), translation-related gene *ribosomal protein L10* (*RPL10*) and *ribosomal protein S13* (*RPS13*), and energy metabolism gene *adenine nucleotide translocator 1* (*ANT1*). Overexpression of mutant ZMIZ1 significantly upregulated all four genes compared to wild-type (Fig. 3g). These findings suggest that p.Ala304Pro activates proliferation-related genes through a gain-of-function mechanism.

### *ZMIZ1* variant disrupts the TGF-β1 signaling pathway

Transforming growth factor-β1 (TGF-β1) signaling plays an indispensable role in early embryonic development, organogenesis, tissue repair and homeostasis. *Small mother against decapentaplegic (SMAD*) is the core transcription factor downstream of TGF-β1. Previous studies demonstrated that ZMIZ1 can interact with SMAD3/4 to enhance the SMAD transcriptional activity, indicating its critical function in TGF-β1-SMAD signaling pathways. To investigate the impact of the p.A304P variant in ZMIZ1 on TGF-β1 signaling pathway, we detected the expression levels of 7 genes in TGF-β1 signaling pathway, including *TGFβ1*, TGF-β1 classical pathway key genes *SMAD2*/*3*/*4*, TGF-β1 non-classical pathway genes *ERK1*/*2*, and *CTGF*, the downstream target gene of TGF-β1. Notably, qPCR results showed that the expression levels of *TGFβ1*, *SMAD3*, *SMAD4* and *CTGF* were significantly changed in the ZMIZ1-WT group compared with the Blank group. However, the mRNA expression levels of *SMAD2*, *ERK2* and *CTGF*, not *TGFβ1*, *SMAD3* and *SMAD4*, were significantly changed in the ZMIZ1-Mutant group compared with the ZMIZ1-WT group (Fig. S3). These results demonstrate that the p.A304P variant in ZMIZ1 disrupts the TGF-β1 signaling pathway, suggesting a potential contribution to NEDDFSA pathogenesis.

### Transcriptional analysis reveals *ZMIZ*1 regulates pathways beyond TGF-β signaling

**Fig. 4:**
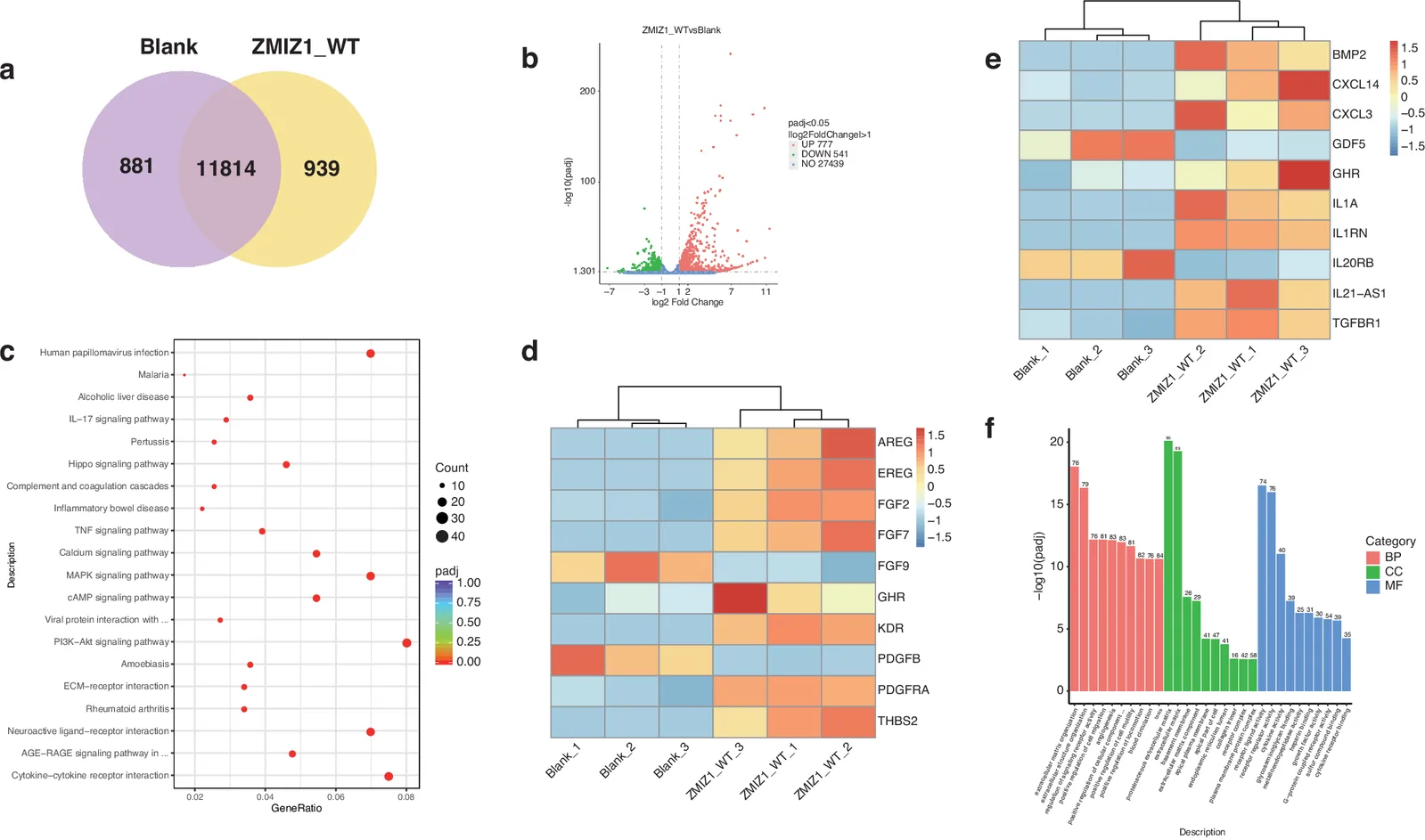
Transcriptome data analysis ZMIZ1-WT group and the Blank group in HSkMCs. **a** Venn diagram showing the distinct and overlapping genes of the transcriptome defined by |log₂FC| ≥ 2 and FDR < 0.05. **b** A total of 1318 differential expressed genes (DEGs) were identified in the ZMIZ1-WT group compared with the Blank group, among which 777 were up-regulated and 541 were down-regulated. **c** Enrichment analysis of KEGG signaling pathway of DEGs showed that there were 56 signaling pathways with significant differences, including the PI3K-Akt signaling pathway and Cytokine-cytokine receptor interaction. **d** Some DEGs associated with the PI3K-Akt signaling pathway between the Blank and ZMIZ1-WT groups are listed. **e** Some DEGs associated with the cytokine-cytokine receptor interaction signaling pathway between the Blank and ZMIZ1-WT groups are listed. **f** GO enrichment analysis for DEGs, which were annotated into three categories: cellular component (CC), molecular function (MF) and biological process (BP). A total of 1007 items were significantly enriched, of which 915 were BP, 15 were CC and 77 were MF.

Previous studies indicate ZMIZ1 modulates both TGF-β and Wnt signaling pathways. To identify major signaling pathways regulated by ZMIZ1 in HSkMCs, we performed transcriptome sequencing comparing vector-transfected (Blank) versus wild-type ZMIZ1-transfected (ZMIZ1-WT) cells. Comparative transcriptomics identified 11,814 shared genes between ZMIZ1-WT and Blank groups (FDR < 0.05, FPKM ≥ 1), with 881 Blank-specific and 939 ZMIZ1-WT-specific differentially expressed genes (|log₂FC| ≥ 2, FDR < 0.05) (Fig. 4a). A total of 1318 DEGs were identified in the ZMIZ1-WT group compared with the Blank group, among which 777 were up-regulated and 541 were down-regulated (Fig. 4b). KEGG enrichment identified 56 significantly altered pathways. Top 20 significantly enriched pathways are presented in Fig. 4c, including the PI3K-Akt signaling pathway (associated with DEGs such as FGF2, FGF7, and THBS2, etc) and Cytokine-cytokine receptor interaction (involving DEGs like CXCL14, CXCL3, and BMP2, etc) (Fig. 4d, e). Further, we conducted GO enrichment analysis for DEGs, which were annotated into three categories: cellular component (CC), molecular function (MF) and biological process (BP). A total of 1007 items were significantly enriched, of which 915 were BP, 15 were CC, and 77 were MF (Fig. 4f).

**Fig. 5:**
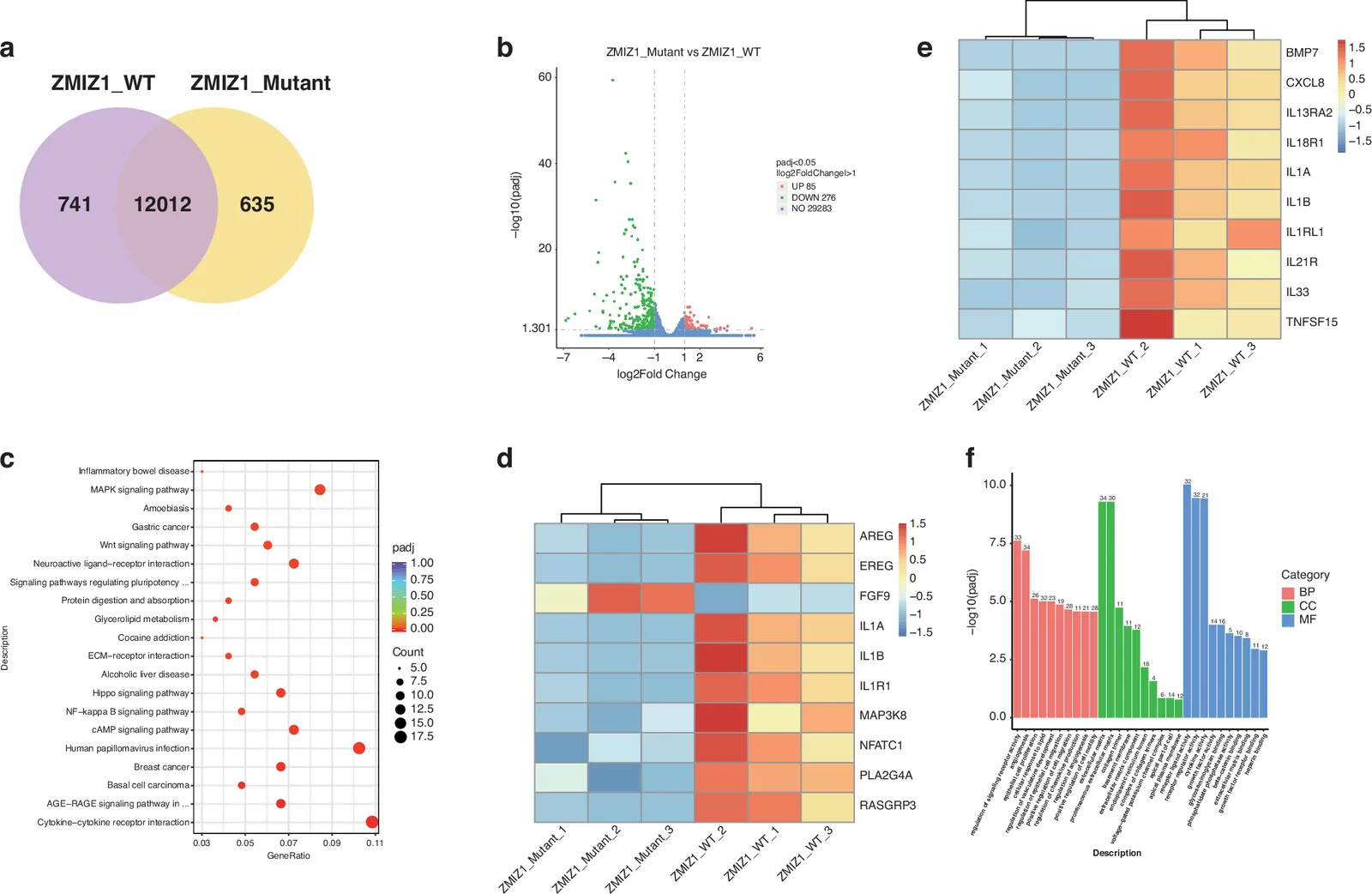
Transcriptome data analysis ZMIZ1-WT group and the Mutant group in HSkMCs. **a** Analysis revealed 12012 genes were commonly expressed in both groups, while 635 genes were uniquely differentially regulated in the ZMIZ1-Mutant group and 741 genes were uniquely differentially regulated in the ZMIZ1-WT group. **b** A total of 361 DEGs were identified in ZMIZ1-mutant group and ZMIZ1-WT group. Among them, 85 genes in the ZMIZ1-mutant group were up-regulated and 276 genes were down-regulated compared with the ZMIZ1-WT group. **c** Enrichment analysis of KEGG signaling pathway for DEGs between the ZMIZ1-mutant group and the ZMIZ1-WT group showed that there were 34 signaling pathways with significant differences. **d** Some DEGs associated with the MAPK signaling pathway between the ZMIZ1-mutant and ZMIZ1-WT groups are listed. **e** Some DEGs associated with the cytokine-cytokine receptor interaction signaling pathway between the ZMIZ1-mutant and ZMIZ1-WT groups were listed. **f** GO enrichment analysis for DEGs, annotated into CC, MF, BP. A total of 539 items were significantly enriched, including 508 of BP, 7 of CC and 24 of MF.

To compare mutant versus wild-type effects, we sequenced ZMIZ1-A304P-transfected (ZMIZ1-Mutant) versus ZMIZ1-WT cells. Analysis revealed 12012 genes were commonly expressed in both groups, while 635 genes were uniquely differentially regulated in the ZMIZ1-mutant group and 741 genes were uniquely differentially regulated in the ZMIZ1-WT group (Fig. 5a). A total of 361 DEGs were identified in ZMIZ1-mutant group and ZMIZ1-WT group. Among them, 85 genes in the ZMIZ1-mutant group were up-regulated and 276 genes were down-regulated compared with the ZMIZ1-WT group (Fig. 5b). KEGG enrichment identified 34 altered pathways. Top significantly enriched pathways are presented in Fig. 5c, including cytokine-cytokine receptor interaction (associated with BMP7, CXL8, IL13RA2, etc) and MAPK signaling pathway (associated with MAP3K8, NFATC, RASGRP3, etc) (Fig. 5d, e). Notably, four pathways were uniquely enriched in Mutant group, the Ras signaling pathway, phospholipase D signaling pathway, basal cell carcinoma, and glycerolipid metabolism. A total of 539 items were significantly enriched, including 508 of BP, 7 of CC and 24 of MF (Fig. 5f). According to the list of DEGs in the ZMIZ1-mutant group compared with the ZMIZ1-WT group, we randomly selected six DEGs for qPCR verification, and the results were consistent with the transcriptome sequencing results (Fig. S4). Collectively, these findings demonstrate ZMIZ1 regulates signaling networks extending beyond TGF-β.

### ZMIZ1 serves as a key regulatory hub for multiple signaling pathways

**Fig. 6:**
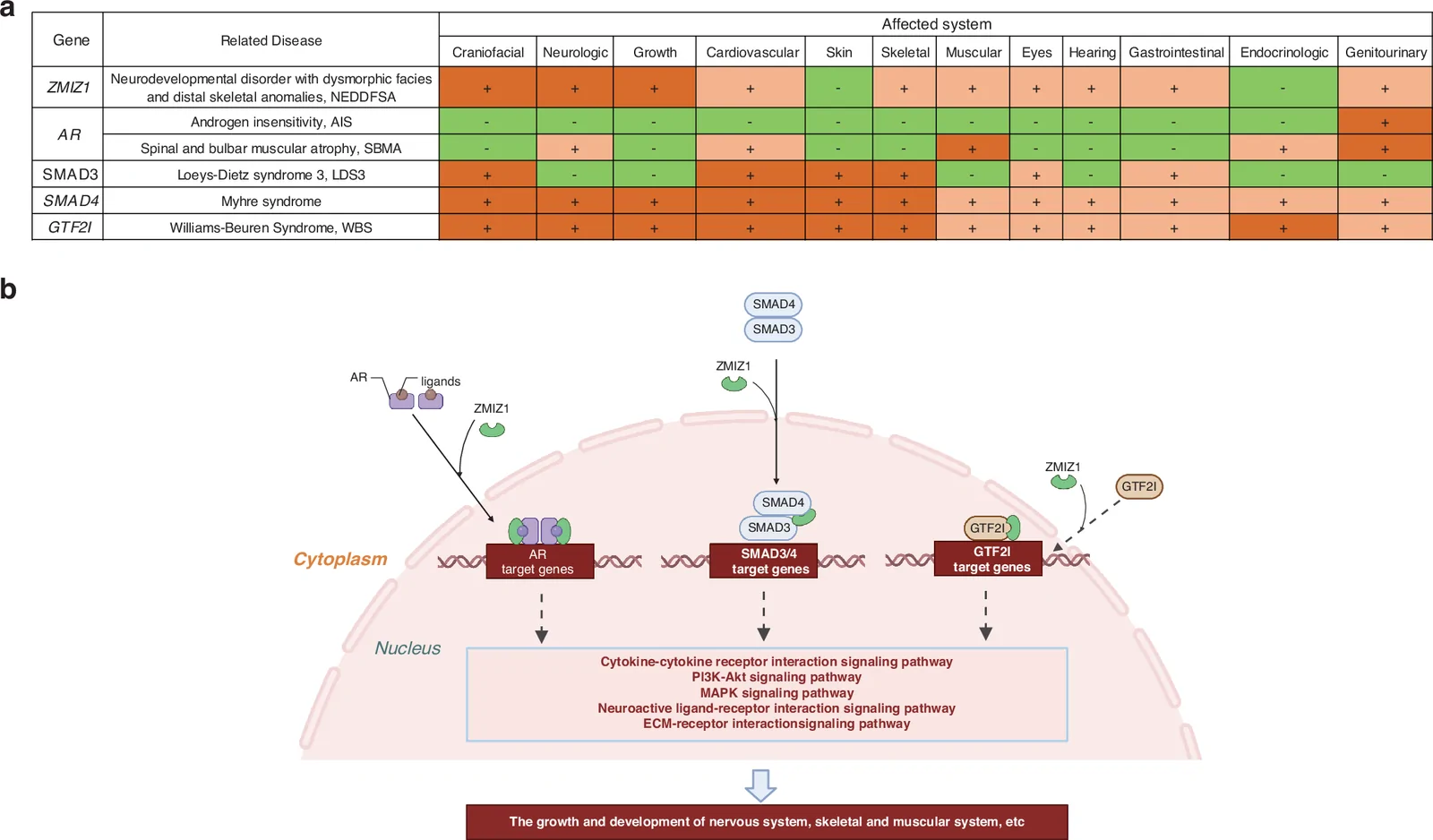
The role of ZMIZ1 in the development of genetic diseases. **a** Genetic diseases and associated phenotypes related to ZMIZ1, androgen receptor (AR), SMAD3/4, and GTF2I are indicated as follows: unaffected is shown in green, while affected is shown in red. The darker the color, the more severe the degree of affection. **b** The schematic diagram illustrates the biological functions of ZMIZ1. ZMIZ1 interacts with AR, SMAD3/4, GTF2I to regulate various signaling pathways.

To elucidate ZMIZ1’s role as a potential transcriptional coactivator, we conducted Co-IP/MS analysis to identify its interaction partners, with particular focus on transcription factor. Beyond known interactors like PCNA and SMARCE1, we discovered several novel candidate proteins, notably general transcription factor IIi (GTF2I) (Table S6). This suggests ZMIZ1 interacts with additional transcription factors beyond the established AR and SMAD3/4 complexes. This interaction, along with previously known interactions with AR, SMAD3/4, and other transcription factors, underscores ZMIZ1’s role as a central regulatory hub in multiple signaling pathways. To determine the roles of ZMIZ1 in genetic diseases in conjunction with AR, SMAD3/4, and GTF2I, we analyzed the genetic diseases and phenotypes caused by mutations in these four genes (Fig. 6a). Results showed that *ZMIZ1* mutations lead to the NEDDFSA, primarily affecting the nervous system, skeletal-muscular system, and reproductive system. *AR* variants are associated with androgen insensitivity syndrome and spinal and bulbar muscular atrophy, mainly impacting the nervous system, muscular system, and reproductive system. *SMAD3/4* mutations are linked to Loeys-Dietz syndrome (LDS), a connective tissue disorder that primarily affects the cardiovascular and skeletal systems. *GTF2I* variants are implicated in Williams-Beuren Syndrome, caused by a deletion in the 7q11.23 region of chromosome 7, and mainly affect the nervous system, skeletal-muscular system, and cardiovascular system. These genetic diseases and phenotypes associated with mutations in ZMIZ1, AR, SMAD3/4, and GTF2I underscore ZMIZ1’s role as a central regulatory hub for multiple signaling pathways.

Our findings suggest that ZMIZ1 orchestrates distinct gene expression patterns and plays a pivotal role in cell growth, development, and tissue repair (Fig. 6b). This regulatory function of ZMIZ1 highlights its importance in maintaining cellular homeostasis and its potential as a therapeutic target for disorders associated with disrupted signaling pathways.

## Discussion

Neurodevelopmental disorder with dysmorphic facies and distal skeletal anomalies (NEDDFSA) is a genetically heterogeneous developmental dysplasia associated with pathogenic variants in *ZMIZ1*.^1^ NEDDFSA is marked by widespread neurodevelopmental delay, early-onset muscle weakness, overall developmental delay, and limited language proficiency, behavioral abnormalities and other prevalent features including joint contracture, eye anomalies, and skeletal deformities affecting the hands and feet.^2,4^ Owing to numerous complications and resemblances to associated diseases, precise clinical diagnosis of neurodevelopmental disorder classifications is difficult through traditional methods, and genetic test assumes a crucial role.^17^

Currently, a total of 27 individuals diagnosed with neurodevelopmental disorders have been identified as harboring pathogenic variants in the *ZMIZ1*.^1–7^ While reported across diverse populations, NEDDFSA appears rare in Chinese cohorts, with merely one case recorded to date.^5^ Among the patients studied, the only exceptions were the c.1491+2T>C variant found in a female from Thailand and the c.2330G>A variant found in a girl from China; all other variants were observed in Caucasian patients residing in the west country.^5^ To the best of our knowledge, this is the second account of *ZMIZ1* variant in Chinese. In this study, we identified a novel missense likely pathogenic variant c.910G>A(p.A304P) in a 11-month-old boy, who exhibited the distinctive clinical symptoms of NEDDFSA, including bilateral auricular deformity, contractures of both hands, congenital vertical talus, developmental delay, hearing impairment, and bilateral cryptorchidism. Integrated bioinformatic and functional analyses classify this variant as likely pathogenic per ACMG guidelines. We fully recognize the limitation of our single-proband study design. To address this, we are currently collaborating with multiple medical institutions to collect additional cases.

*ZMIZ1* (previously *ZIMP10*, *RAI17*, or *KIAA1224*) was first isolated from a fetal brain cDNA library by Nagase et al. in 1999.^10^ Tissue-specific expression analyses reveal broad distribution (heart, thyroid, immune cells, ovaries, retina, brain), with highest levels in the pineal gland.^18^ The encoded protein functions as a transcriptional co-activator and belongs to the PIAS (Protein Inhibitor of Activated STAT) family.^9^ Notably, *ZMIZ1* possesses a conserved MIZ domain, which serves a pivotal function in protein-protein interaction and SUMOylation.^19^ According to previous reports, *ZMIZ1* has been implicated in the regulation of various transcription regulators, encompassing AR, SMAD3, SMAD4, and p53. In the Drosophila melanogaster, tonalli (tna), an ortholog of ZMIZ1, was discovered and found to interact with ATP-dependent SWI/SNF complexes, signifying a possible participation in the process of chromatin remodeling. The *ZMIZ1* gene is recently found to interact with BRG1 (SMARCA4), BAF57 (SMARCE1), and SATB1 to govern chromatin remodeling in humans.^20–24^ Notably, our study demonstrates significantly upregulated expression of *SMARCA4*, *RPL10*, *RPS13*, and *SLC25A4* in ZMIZ1 p.A304P overexpressed cells, confirming that the ZMIZ1 can control the expression levels of genes related to chromosome remodeling, ribosome components, and energy metabolism synthesis.

The protein-protein interaction network and Gene Ontology (GO) analysis revealed profoundly enrichment in mesenchyme/tube morphogenesis, hypoxia, transcription regulation, response to stimulus, epithelial to mesenchymal transition, endocardium development, and the morphogenetic process of the cardiac left ventricle.^5^ Network members localized predominantly in the nucleus, forming distinct complexes that modulate target genes expression. *ZMIZ1* is crucial for embryonic development, exhibiting dynamic expression patterns within various tissues, including neural tissues, craniofacial structures, the mandible, foregut, limb buds, optic vesicle, otic pit, and somites, at distinct stages of development in mice embryos.^1,25^ This expression pattern aligns precisely with clinical manifestations in ZMIZ1-mutant patients.

The results demonstrated that there were significant changes in the expression levels of TGFβ1, SMAD3, SMAD4 and CTGF between the ZMIZ1-WT group and the Blank group. These confirm that the *ZMIZ1* play a crucial role in the progression of fibrosis by involved in TGF-β1 signaling pathway. This expands the underlying mechanism by which the *ZMIZ1* pathogenic variant exerts its influence on the physiological development of various systems, including the nervous, joint, and skeletal systems. Notably, the difference of mRNA expression levels of *SMAD2*, *ERK2* and *CTGF* in the ZMIZ1-mutan group compared with the ZMIZ1-WT group suggesting changes in function after ZMIZ1 p.A304P variant.

To date, 21 variants in the *ZMIZ1* gene have been reported in the NEDDFSA patients, including 9 missense variants, 2 splice variants, 7 frameshift variants, 3 balanced translocations (Table 1). Among the 9 missense variants, half were in the alanine-rich sequence. Coincidentally, our patient’s variant is also located in this region. Situated within the intrinsically disordered N-terminal part of ZMIZ1 is the alanine-abundant low-complexity regions (LCR).^26^ These sequences were remarkably preserved across various species throughout evolution, indicating their significance for the optimal function of ZMIZ1. It has been reported that numerous transcription factors or transcriptional mediators may engage with ZMIZ1 at the transcription initiation sites through their strikingly LCRs to create localized phase-separated condensates (also referred to as droplets) that enhance DNA binding, attract RNA polymerase II (RNA Pol II), and initiate transcription.^26,27^ These unique condensates were characterized by a balance between optimal functionality and the potential for abnormal aggregation. Many diseases are caused by faulty phase transitions occurring within droplets that have liquid-like properties. Based on IUPred2A analysis, ZMIZ1 features three low-complexity regions: one that is rich in alanine and two segments rich in proline, potentially critical for its phase separation and transcriptional mediator activity.^28,29^ As anticipated, the variants may alter phosphorylation patterns within the alanine-rich region, potentially impacting local structural conformation. The potential molecular mechanism for the variant hotspot of *ZMIZ1* being the alanine-rich region remains to be experimentally confirmed. Interestingly, the novel p.A304P variant identified in this study is the first of all that have been observed to alter the subcellular localization of ZMIZ1.

**Table 1:** *ZMIZ1* variants reported in the 28 NEDDFSA patients.

	Subject 1	Subject 2	Subject 3	Subject 4	Subject 5	Subject 6	Subject 7	Subject 8
Origin	France	USA	USA	USA	France	USA	USA	USA
Variant type	Missense mutation	Missense mutation	Frameshift mutation	Missense mutation	Missense mutation	Frameshift mutation	Missense mutation	Missense mutation
Variant position	exon 11	exon 11	exon 25	exon 11	exon 11	exon 23	exon 7	exon 22
cDNA Change	c.899C>T	c.859G>A	c.3112dupA	c.899C>T	c.893C>T	c.2752dupC	c.272A>G	c.2610C>T
Protein Alteration	p.Thr300Met	p.Ala287Thr	p.Thr1038Asnfs*4	p.Thr300Met	p.Thr298Ile	p.Gln920Profs *34	p.Lys91Arg	p.Ser870Ser
Gender	M	F	F	F	M	F	F	M
Age	11 y, 8 m	12 y, 3 m	12 y, 3 m	1 y, 6 m	5 y, 9 m	20 y	3 y	6 y, 7 m
Growth failure	N	Y	N	NA	Y	Y	NA	N
Intellectual disability	Y	Y	Y	Y	Y	Y	Y	Y
Motor delay	Y	N	N	Y	Y	N	Y	Y
Speech delay	Y	N	N	NA	Y	Y	Y	Y
Facial dysmorphism	Y	Y	Y	Y	Y	N	NA	Y
Joint hypermobility	N	Y	N	Y	N	N	NA	Y
hearing loss	N	N	Y	N	Y	N	N	N
Congenital Malformations	N	Y	Y	Y	Y	Y	N	Y
Feed difficulties	N	N	NA	Y	Y	Y	Y	NA
REFERENCES	Am J Hum Genet. 2019	Am J Hum Genet. 2019	Am J Hum Genet. 2019	Am J Hum Genet. 2019	Am J Hum Genet. 2019	Am J Hum Genet. 2019	Am J Hum Genet. 2019	Am J Hum Genet. 2019

	Subject 9	Subject 10	Subject 11	Subject 12	Subject 13	Subject 14	Subject 15	Subject 16
Origin	USA	UK	USA	Poland	USA	USA	USA	France
Variant type	Missense mutation	Frameshift mutation	Splicing mutation	Frameshift mutation	Frameshift mutation	Frameshift mutation	Frameshift mutation	Missense mutation
Variant position	exon 11	exon 13	Intron 24	exon 13	exon 13	exon 13	exon 13	exon 11
cDNA Change	c.887C> A	c.1386dupC	c.3097-2A>G	c.3021delC	c.1386dupC	c.1386dupC	c.1386dupC	c.887C>A
Protein Alteration	p.Thr296Lys	p.Thr463Hisfs *14	p.?	p.Phe1008Leufs*7	p.Thr463Hisfs *14	p.Thr463Hisfs *14	p.Thr463Hisfs *14	p.Thr296Lys
Gender	F	F	M	M	F	M	M	F
Age	8 y, 6 m	19 y	1 y, 4 m	3 y, 6 m	7 y	10 y	11 y	7 y
Growth failure	Y	Y	N	N	Y	Y	Y	Y
Intellectual disability	Y	Y	Y	Y	Y	Y	Y	Y
Motor delay	Y	Y	Y	Y	NA	NA	NA	Y
Speech delay	Y	Y	Y	Y	Y	Y	NA	Y
Facial dysmorphism	Y	Y	Y	Y	Y	Y	Y	Y
Joint hypermobility	N	Y	Y	N	Y	N	Y	Y
hearing loss	N	N	N	N	Y	N	N	Y
Congenital Malformations	Y	Y	Y	Y	Y	Y	Y	N
Feed difficulties	Y	Y	Y	Y	N	N	N	Y
REFERENCES	Am J Hum Genet. 2019	Am J Hum Genet. 2019	Am J Hum Genet. 2019	Am J Hum Genet. 2019	Am J Hum Genet. 2019	Am J Hum Genet. 2019	Am J Hum Genet. 2019	Am J Hum Genet. 2019

	Subject 17	Subject 18	Subject 19	Subject 20	Subject 21	Subject 22	Subject 23	Subject 24
Origin	USA	France	France	China	USA	USA	USA	Thailand
Variant type	Frameshift mutation	Balanced translocation	Balanced translocation	Missense mutation	Frameshift mutation	Frameshift mutation	Frameshift mutation	Splicing mutation
Variant position	exon 23	-	-	exon 20	exon 13	exon 13	exon 13	Intron 14
cDNA Change	c.2835delT	t(X;10)	t(10,12)	c.2330G>A	c.1310delC	c.1310delC	c.1310delC	c.1491+2 T > C
Protein Alteration	p.Met946Cysfs*61	(q27.3;q22.3)	(q22.2;q24.3)	p.Gly777Glu	p.Pro437ArgfsX84	p.Pro437ArgfsX84	p.Pro437ArgfsX84	ND
Gender	F	M	F	F	M	M	M	M
Age	12 y, 17 m	17 y	2 y,9 m	5 y	7 y	12 y	53 y	5 y
Growth failure	Y	N	N	Y	Y	Y	NA	Y
Intellectual disability	Y	Y	Y	Y	Y	Y	Y	Y
Motor delay	NA	Y	Y	N	Y	Y	NA	Y
Speech delay	Y	Y	Y	2 y	Y	Y	Y	Y
Facial dysmorphism	Y	N	N	Y	Y	Y	Y	Y
Joint hypermobility	N	Y	N	Y	NA	NA	NA	NA
hearing loss	N	N	N	N	Y	NA	NA	N
Congenital Malformations	Y	Y	N	Y	Y	Y	Y	N
Feed difficulties	N	N	N	N	Y	Y	NA	Y
REFERENCES	Am J Hum Genet. 2019	Am J Hum Genet. 2019	Am J Hum Genet. 2019	Front Genet. 2022	Am J Med Genet A. 2020	Am J Med Genet A. 2020	Am J Med Genet A. 2020	Genes (Basel). 2021

	Subject 25	Subject 26	Subject 27	Subject 28
Origin	México	Sweden	Canada	China
Variant type	Balancedtranslocation	Frameshiftmutation	Missense variant	Missensemutation
Variant position	-	exon 25		exon 11
cDNA Change	t(10;19)	c.3113_3114del	c.881C>T	c.910G>C
Protein Alteration	(q22.3;q13.33)	p.Thr1038Lysfs*3	p.Thr294Ile	p.Ala304Pro
Gender	F	M	M	M
Age	11 y	2.5 y	2 y	1 y
Growth failure	N	Y	NA	Y
Intellectual disability	Y	Y	Y	NA
Motor delay	NA	Y	NA	Y
Speech delay	Y	Y	Y	NA
Facial dysmorphism	Y	Y	Y	Y
Joint hypermobility	Y	NA	NA	Y
hearing loss	NA	NA	NA	N
Congenital Malformations	NA	Y	Y	Y
Feed difficulties	NA	Y	Y	N
REFERENCES	Neurogenetics. 2015	Journal of Pediatric Surgery Case Reports,2021	Ophthalmic Genetics, 2024	this study

*M* male, *F* female, *N* no, *Y* yes, *y* years, *m* months, *NA* no available.

NEDDFSA patients exhibit not only abnormal nervous system development but also potential involvement of muscle and bone cells. Joint hypermobility, a distinct joint feature of NEDDFSA, caught our eyes. In vitro, we demonstrated for the first time that the p.A304P variant in ZMIZ1 promotes proliferation, migration, and invasion of HSkMC cells, as evidenced by EdU incorporation, scratch wound healing, and transwell migration assays. RNA-seq analysis show that a total of 1318 DEGs, 777 up-regulated and 541 down-regulated, were identified in the ZMIZ1-WT group compared with the Blank group. 56 KEGG signaling pathway of DEGs were significant differences, including the Cytokine-cytokine receptor interaction, PI3K-Akt signaling pathway, and Neuroactive ligand-receptor interaction. A total of 361 DEGs were identified in ZMIZ1-mutant group and ZMIZ1-WT group. 34 KEGG signaling pathway were significant differences. Among them, the Ras signaling pathway, phospholipase D signaling pathway, basal cell carcinoma, and glycerolipid metabolism were not significantly enriched in ZMIZ1-WT group compared with Blank group. Based on the above results, we speculated that *ZMIZ1* gene plays an important role in regulating cell proliferation by Cytokine-cytokine receptor interaction. It is noteworthy that the Cytokine-cytokine receptor interaction signaling pathway is also significantly regulated when WT or mutant *ZMIZ1* is overexpressed. This suggests that the function of the *ZMIZ1* gene needs to be more in-depth research, especially its role in cell proliferation and immune response.

## Conclusion

In conclusion, our study identified a novel likely pathogenic variant c.910G>C (p.A304P) in the *ZMIZ1* associated with NEDDFSA in a Chinese patient. We demonstrated the pathogenicity of this variant and provided insights into the cellular and physiological mechanisms underlying NEDDFSA, particularly in muscle cells. For the first time, we identified a new transcription factor, GTF2I, that interacts with ZMIZ1, further highlighting its role as a central regulatory hub in multiple signaling pathways. Importantly, our study revealed that the regulatory signaling pathway of ZMIZ1 extends beyond the TGF-β signaling pathway.

Further studies are needed to understand the detailed mechanisms by which ZMIZ1 modulates these diverse signaling pathways and to explore potential therapeutic targets for NEDDFSA. The identification of this novel ZMIZ1 variant and its functional impact on cellular processes underscores the importance of continued investigation into the genetic and cellular basis of NEDDFSA. Our study not only expands the ZMIZ1 mutational spectrum but also highlights the need for a deeper understanding of the intricate signaling networks regulated by ZMIZ1 in various cell types and tissues.

## Supplementary information

supplementary figure

Table S1

Table S2

Table S3

Table S4

Table S5

Table S6

## Data Availability

The datasets generated for this study are available in online repositories. The names of the repository/repositories and accession number(s) can be found at: https://www.ncbi.nlm.nih.gov/genbank/, NM_020338.3.
