# Supplementary material for: Genetic and functional analysis of ZMIZ1 in neurodevelopmental disorder with dysmorphic facies and distal skeletal anomalies (NEDDFSA): insights from muscle cells and signaling pathways
Source: Pediatr Res. 2025 Dec 7;100(2):872–84. doi: 10.1038/s41390-025-04612-x (PMC13638472; doi:10.1038/s41390-025-04612-x)
Supplement: Supplementary file 1 [file 41390_2025_4612_MOESM1_ESM.pdf]

Figure S1. The workflow diagram of this study

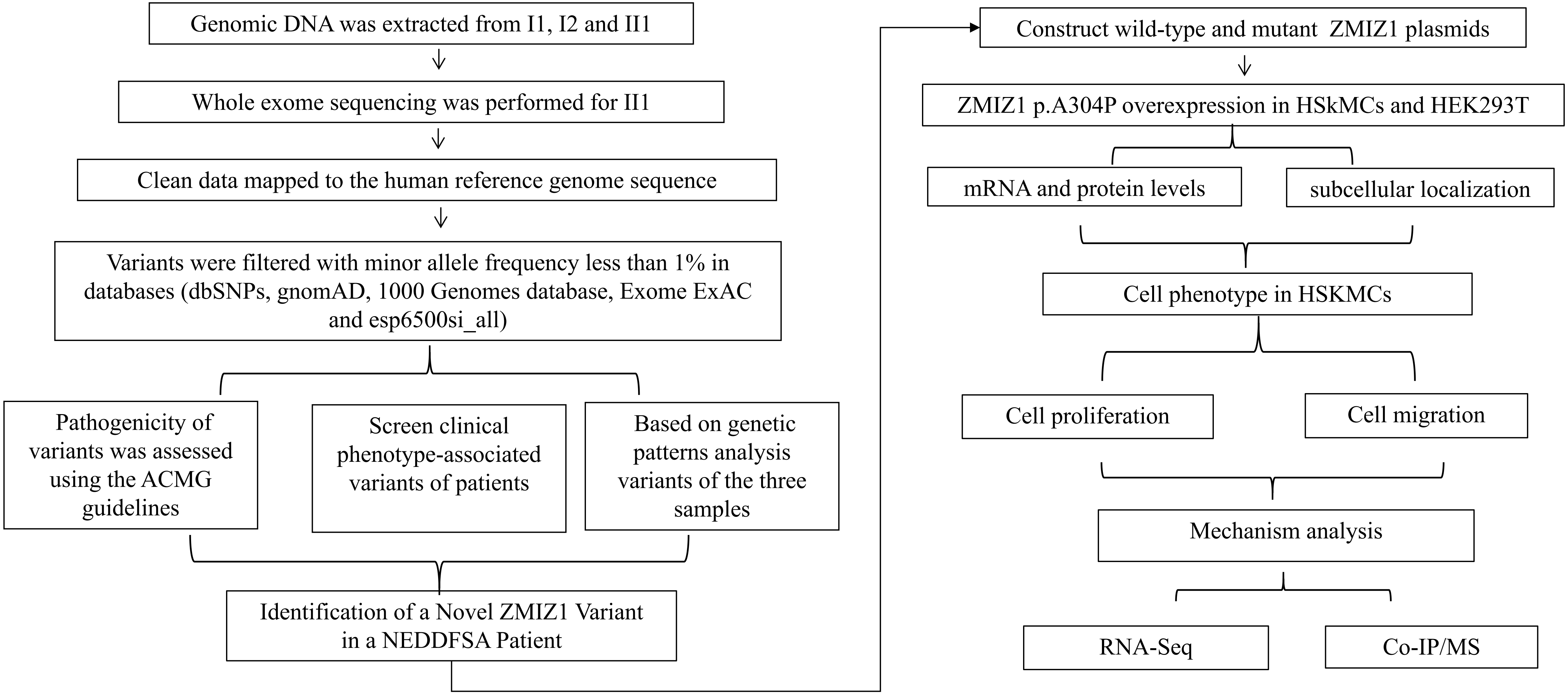

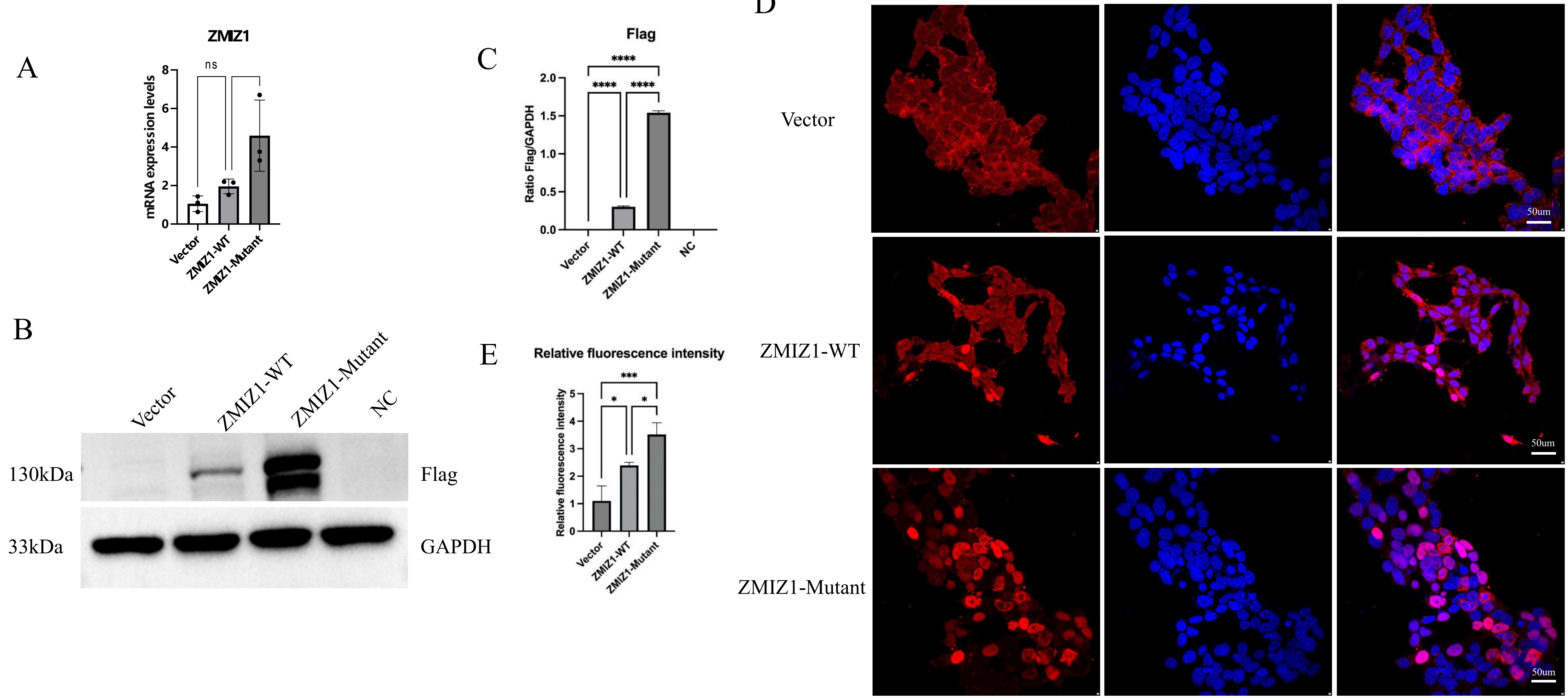

Figure S2. Function and expression of ZMIZ1 variant p.A304P in HEK293T.(A) Expression of ZMIZ1 mRNA was assessed by qRT-PCR in HEK293T. (B-C) The protein expression levels of ZMIZ1 were measured by Western blot. (D-E) Cellular localization of ZMIZ1 in cells transfected with plasmids pcDNA3.1, pcDNA3.1-ZMIZ1-WT, or pcDNA3.1-ZMIZ1-mutant. Cells were stained with anti-ZMIZ1 antibody. Nuclei were stained with DAPI (Blue). ZMIZ1-WT was diffusely expressed in the nucleus and cytoplasm, while ZMIZ1-mutant was expressed mainly in the nucleus. Scar bar: 50um.

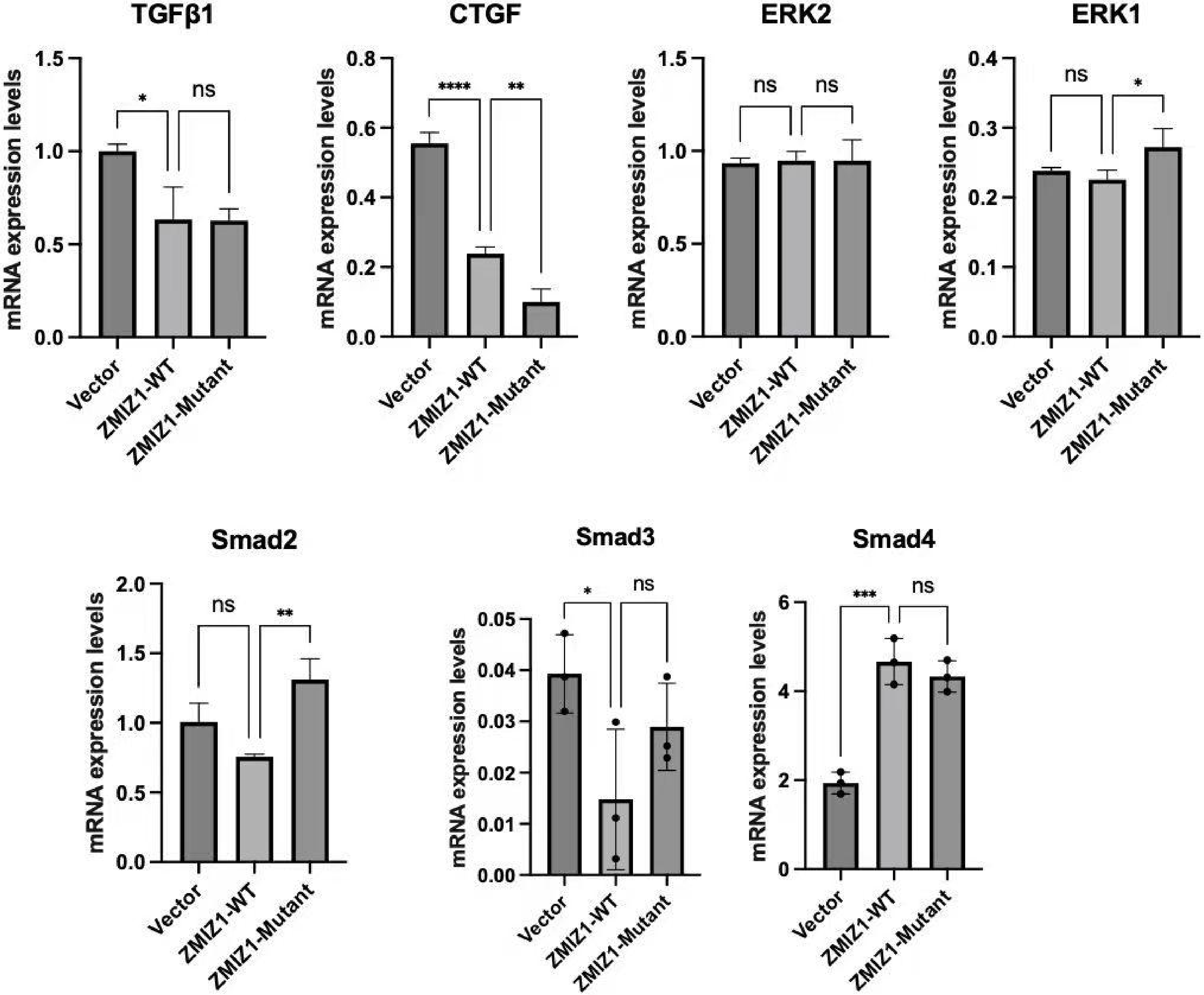

Figure S3. ZMIZ1 variant disrupts the transforming growth factor-β1 signaling pathways

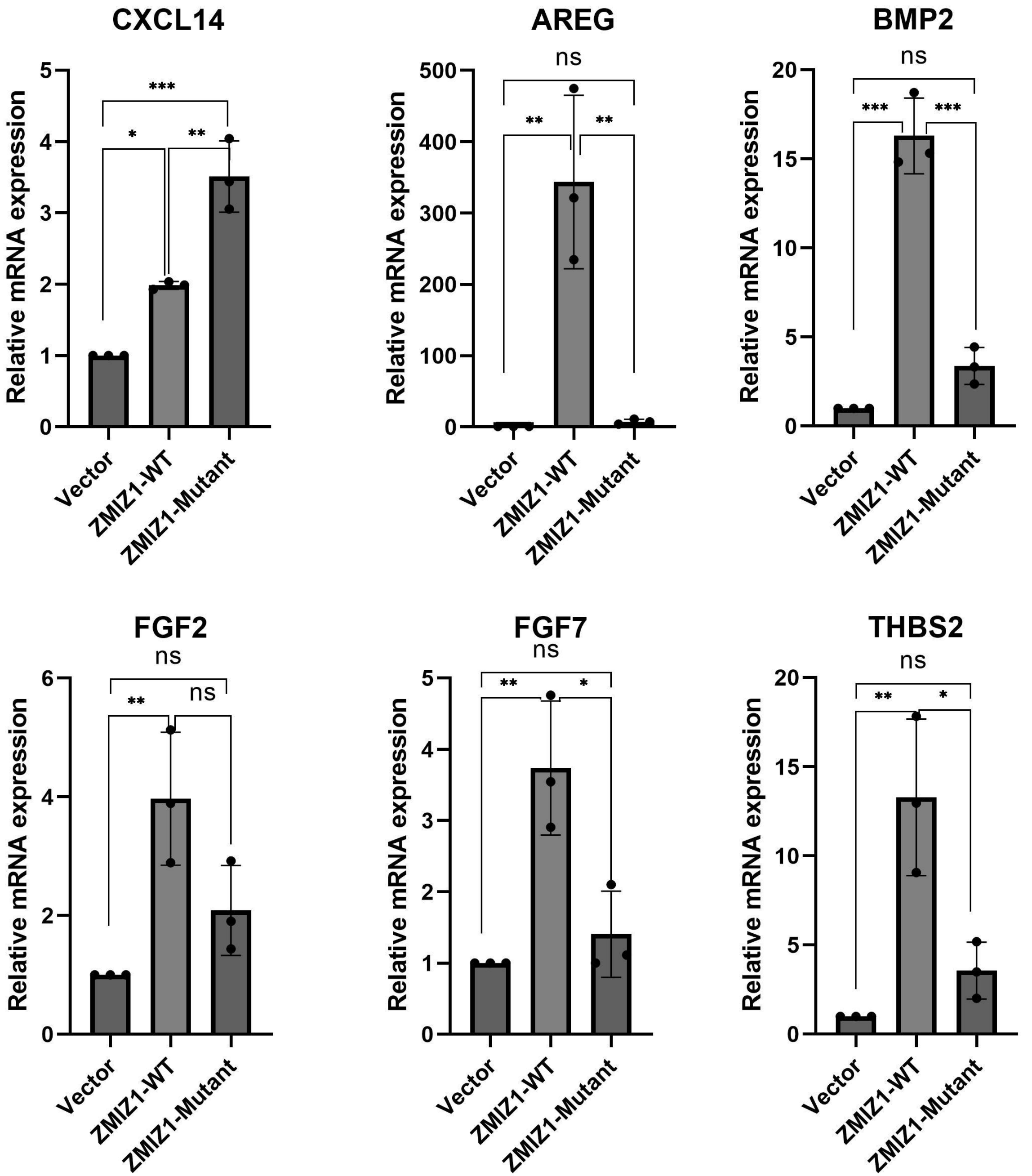

Figure S4. Expression of mRNA was assessed by qRT-PCR. Error bars represent the SD of four independent experiments.
