# Supplementary material for: Genetic and functional analysis of ZMIZ1 in neurodevelopmental disorder with dysmorphic facies and distal skeletal anomalies (NEDDFSA): insights from muscle cells and signaling pathways
Source: Pediatr Res. 2025 Dec 7;100(2):872–84. doi: 10.1038/s41390-025-04612-x (PMC13638472; doi:10.1038/s41390-025-04612-x)
Supplement: Supplementary file 2 [file 41390_2025_4612_MOESM2_ESM.pdf]

Table S1. Quality control data of whole exome sequencing of the patient

| Sample | II1 |
|---|---|
| Clean bases | 13974849995 |
| Clean reads | 93766094 |
| Mapped reads | 93510225(99.73%) |
| Duplicate reads | 20521872(21.95%) |
| Unique reads | 72988353(78.05%) |
| Reads uniquely mapped to target | 47616508 |
| Reads uniquely mapped to genome | 67070497 |
| Raw reads | 106188338 |
| Raw data(G) | 15.9282507 |
| Q20(%) | 96.40% |
| Q30(%) | 91.04% |
| GC(%) | 51.50% |
| Total bases on target | 39086460 |
| Total bases near target | 72508023 |
| Total sequences on target(Mb) | 5181.7 |
| Total sequences near target(Mb) | 3488.37 |
| Total effective yield(Mb) | 10453.47 |
| Fraction of effective bases on target | 49.57% |
| Fraction of effective bases on or near target | 82.94% |
| Fraction of uniquely mapped on target | 70.99% |
| Average sequencing depth on target | 132.57 |
| Average sequencing depth near target | 48.11 |
| Average insert size of the library | 290.88 |
| Base covered on target | 38839380 |
| Coverage of target region | 99.37% |
| Base covered near target | 72378147 |
| Coverage of near target | 99.80% |
| Mismatch rate in target | 0.40% |
| Mismatch rate in total | 0.34% |
| Fraction of target covered at least 4x | 99.33% |
| Fraction of target covered at least 10x | 99.26% |
| Fraction of target covered at least 20x | 98.97% |
| Fraction of target covered at least 30x | 98.18% |
| Fraction of target covered at least 50x | 93.35% |
