# Supplementary material for: Genetic and functional analysis of ZMIZ1 in neurodevelopmental disorder with dysmorphic facies and distal skeletal anomalies (NEDDFSA): insights from muscle cells and signaling pathways
Source: Pediatr Res. 2025 Dec 7;100(2):872–84. doi: 10.1038/s41390-025-04612-x (PMC13638472; doi:10.1038/s41390-025-04612-x)
Supplement: Supplementary file 3 [file 41390_2025_4612_MOESM3_ESM.pdf]

Table S2.primers used for variants amplification and qPCR

Primer name	Primer (5'-3')
ZMIZ1-F1	GATGGAGCTGGAGTGAGGTG
ZMIZ1-R1	AGGTTCTACCCGAGAGCCAT
SLC25A4-F1	AGCGTGAGTTCCATGGTCTG
SLC25A4-R1	GCCTTGGACAGAGACGTTGA
RPL10-F1	GGGGCTGACAGATCCACATC
RPL10-R1	CGCTTTTCAGCCACCATGTC
RPS13-F1	TCCCAGTCGGCTTTACCCTA
RPS13-R1	GCTCCTTCACGTCGTCAGAT
TGFβ1-F1	GAGCCCTGGACACCAACTAT
TGFβ1-R1	CAGAAGTTGGCATGGTAGCC
CTGF-F1	ATTAGAGCCAACTGCCTGGT
CTGF-R1	AGGAGGCGTTGTCATTGGTA
Smad2-F1	GCAGGAATTGAGCCACAGAG
Smad2-R1	TGGAGAGCCTGTGTCCATAC
Smad3-F1	AAGATCCCACCAGGATGCAA
Smad3-R1	GGTCAACTGGTAGACAGCCT
SMAD4-F1	GACCCTTCTGGAGGAGATCG
SMAD4-R1	ACCTTGCTCTCTCAATGGCT
ERK1-F1	CATCGGCATCCGAGACATTC
ERK1-R1	TCCATCAGGTCCTGCACAAT
ERK2-F1	ACCAACCTCTCGTACATCGG
ERK2-R1	TAGGTCTGGTGCTCAAAGGG
SMARCA4-F1	GCTCAAGGCCATCGAGGAG
SMARCA4-R1	TAGGGGAGAGTTTCTCGGCA
