# Supplementary material for: Genetic and functional analysis of ZMIZ1 in neurodevelopmental disorder with dysmorphic facies and distal skeletal anomalies (NEDDFSA): insights from muscle cells and signaling pathways
Source: Pediatr Res. 2025 Dec 7;100(2):872–84. doi: 10.1038/s41390-025-04612-x (PMC13638472; doi:10.1038/s41390-025-04612-x)
Supplement: Supplementary file 4 [file 41390_2025_4612_MOESM6_ESM.pdf]

Table S5 Variant assessment of a novel *ZMIZ1* variant NM_020338.3: c.910G>C; p.Ala304Pro) according to the ACMG/AMP guidelines.

ACMG/AMP criteria	Criteria description	Evidence fulfilled by the ZMIZ1 variant	Points if criteria met
PATHOGENIC CRITERIA			
PM2: Pathogenic, Supporting Evidence	Absent from controls in Exome Sequencing Project, 1000 Genomes Project, Exome Aggregation Consortium, or gnomAD.	The variant is completely absent from gnomAD	1
PS2: Pathogenic, Moderate Evidence	De novo (both maternity and paternity confirmed) in a patient with the disease and no family history	The Trio-WES analysis results indicate that both parents of the proband do not have this mutation and have no family history	2
PP3: Pathogenic, Supporting Evidence	Multiple lines of computational evidence support a deleterious effect on the gene or gene product (conservation, evolutionary, splicing impact, etc.).	Multiple software such as SIFT, Polyphen2, Mutation Taster predict harmful effects	1
PS3: Pathogenic Moderate Evidence	Well-established in vitro or in vivo functional studies sup portive of a damaging effect on the gene or gene product	Our study demonstrates that this mutation le ads to a 20% increase in gene expression in 293T and affects the TGF - β signaling path way	2
BENIGN CRITERIA			
None met			0
Total points for evidence in support of variant being benign			0
Variant score (Pathogenic evidence total score - Benign evidence total score)			6

Likely Pathogenic (4 points with no benign criteria being met). A score of 0-6 is variants of uncertain significance. A score of 6–9 is likely pathogenic. A score of ≥10 is pathogenic.

ACMG/AMP The American College of Medical Genetics and Genomics-Association for Molecular Pathology, *CGD* clinical genomic database, *GenCC* the Gene Curation Coalition, *Mondo* the Mondo Disease Ontology, *OMIM* online Mendelian Inheritance in Man.
